# Increased Wnt/β-catenin signaling contributes to autophagy inhibition resulting from a dietary magnesium deficiency in injury-induced osteoarthritis

**DOI:** 10.1186/s13075-022-02848-0

**Published:** 2022-07-08

**Authors:** Ruijun Bai, Michael Z. Miao, Hui Li, Yiqing Wang, Ruixue Hou, Ke He, Xuan Wu, Hongyu Jin, Chao Zeng, Yang Cui, Guanghua Lei

**Affiliations:** 1 Department of Orthopaedics, Xiangya Hospital, Central South University, 87 Xiangya Road, Changsha, Hunan China; 2 Thurston Arthritis Research Center, Division of Rheumatology, Allergy, and Immunology, School of Medicine, University of North Carolina at Chapel Hill, Chapel Hill, NC USA; 3 Curriculum in Oral and Craniofacial Biomedicine, Adams School of Dentistry, University of North Carolina at Chapel Hill, Chapel Hill, NC USA; 4 Clinical and Translational Epidemiology Unit, Massachusetts General Hospital and Harvard Medical School, Boston, MA USA; 5 Division of Gastroenterology, Massachusetts General Hospital and Harvard Medical School, Boston, MA USA; 6 Department of Population Health Science and Policy, Icahn School of Medical at Mount Sinai, New York, NY USA; 7 Hunan Key Laboratory of Joint Degeneration and Injury, Changsha, China; 8 Hunan Engineering Research Center of Osteoarthritis, Changsha, China; 9 National Clinical Research Center for Geriatric Disorders, Xiangya Hospital, Central South University, Changsha, China; 10 Xiangya International Medical Center, Xiangya Hospital, Central South University, 87 Xiangya Road, Changsha, 410008 Hunan China

**Keywords:** Dietary magnesium, Autophagy, Wnt/ β-catenin, Osteoarthritis

## Abstract

**Background:**

Dietary magnesium deficiency, which is common in modern diet, has been associated with osteoarthritis (OA) susceptibility. Despite this clinical association, no study has addressed if dietary magnesium deficiency accelerates OA development, especially at molecular level. This study aimed to explore aggravating effects of dietary magnesium deficiency on cartilage damage in an injury-induced murine OA model and to determine the underlying mechanism.

**Methods:**

Twelve-week-old C57BL/6J mice subject to injury-induced OA modeling were randomized into different diet groups in which the mice were fed a diet with daily recommended magnesium content (500 mg/kg) or diets with low magnesium content (100 or 300 mg/kg). Articular cartilage damage was evaluated using the OARSI score. To determine molecular mechanisms in vitro, mouse chondrocytes were treated with media of low magnesium conditions at 0.1 and 0.4 mM, compared with normal magnesium condition at 0.7 mM as control. Anabolic and catabolic factors, autophagy markers, β-catenin, Wnt ligands, and a magnesium channel transient receptor potential cation channel subfamily member 7 (TRPM7) were analyzed by quantitative real-time PCR and immunoblotting. Autolysosomes were detected by DALGreen staining via fluorescence microscopy and autophagosomes were evaluated by transmission electron microscopy. Autophagy markers, β-catenin, and TRPM7 were assessed in vivo in the mouse cartilage, comparing between dietary magnesium deficiency and normal diet, by immunohistochemistry.

**Results:**

Dietary magnesium deficiency aggravated injury-induced cartilage damage, indicated by significant higher OARSI scores. Autophagy markers LC3-II and Beclin-1 were decreased both in low magnesium diet-fed mice and low magnesium-treated chondrocytes. The number of autolysosomes and autophagosomes was also reduced under low magnesium conditions. Moreover, magnesium deficiency induced decreased anabolic and increased catabolic effect of chondrocytes which could be restored by autophagy activator rapamycin. In addition, reduced autophagy under low magnesium conditions is mediated by activated Wnt/β-catenin signaling. The expression of TRPM7 also decreased in low magnesium diet-fed mice, indicating that downstream changes could be regulated through this channel.

**Conclusions:**

Dietary magnesium deficiency contributes to OA development, which is mediated by reduced autophagy through Wnt/β-catenin signaling activation. These findings indicated potential benefits of adequate dietary magnesium for OA patients or those individuals at high risk of OA.

**Supplementary Information:**

The online version contains supplementary material available at 10.1186/s13075-022-02848-0.

## Background

Osteoarthritis (OA) is a complex degenerative joint disease that affects mostly middle-aged and older adults and involves progressive deterioration of articular cartilage [1]. The existing treatments for OA can only mitigate the symptoms without altering the underlying disease process [2]. Thus, exploring pathophysiology and therapeutic targets of OA is sorely needed. Several studies have shown that various dietary micronutrients deficiency is associated with the development of OA, and some dietary micronutrient supplements may play a protective role in OA [3–5], indicating that dietary micronutrient can be a potential therapeutic target for OA prevention and treatment.

Magnesium (Mg), one of the most important micronutrients, is the second-most abundant intracellular and fourth-most abundant extracellular cation in the human body [6, 7]. As a cofactor in more than 300 enzymatic reactions, magnesium is essential for many intracellular physiological functions [8]. Previous studies [7, 9–12] have reported a significant lower serum magnesium concentration or dietary magnesium intake in OA patients. However, whether there is a causal relationship between magnesium deficiency and OA still remains unknown. Additionally, since magnesium is predominantly obtained from the diet through green leafy vegetables and unprocessed grains [13], the modern diet, especially the Western dietary pattern, has been rich in refined foods, causing low dietary magnesium intake to become quite common [14]. Therefore, illuminating the effect of dietary magnesium deficiency on the development of OA may help uncover new insights into pathophysiology of OA and offer beneficial recommendations for those with inadequate daily magnesium intake.

As an important intracellular process maintaining cell homeostasis, autophagy is one of the key factors in the etiology of OA [15]. Recent studies showed that autophagy markers were decreased in human OA, as well as aging-related and surgery-induced OA mouse cartilage [16]. It has been further confirmed that intra-articular injection of an autophagy activator alleviated cartilage damage in an experimental OA model [17, 18]. In addition, autophagy is greatly influenced by magnesium, but the effect of magnesium on autophagy is quite different across distinct cell types. For example, autophagy was greatly induced in bladder cancer cells [19] but was inhibited in bone marrow mesenchymal stem cells [20–22] by magnesium. To date, whether autophagy is involved in magnesium deficient chondrocytes has not been elucidated.

Wnt/β-catenin signaling pathway plays a crucial role in tissue repair and joint homeostasis [23, 24]. Appropriate levels of Wnt/β-catenin are necessary for the survival of chondrocytes [23, 25, 26], while over-activated Wnt/β-catenin pathway promotes chondrocyte differentiation and hypertrophy, accompanied by decreased anabolic and increased catabolic activity of articular cartilage, resulting in OA development [27, 28]. Hence, Wnt/β-catenin pathway should be tightly regulated and maintained at a proper level for cartilage homeostasis. Furthermore, previous studies have shown that the Wnt/β-catenin pathway can be activated by magnesium deficiency in vascular smooth muscle cells [29, 30]. Nonetheless, whether similar phenomenon exists in chondrocytes is still not clear.

Based on the evidence above, we hypothesized that reduced autophagy regulated by Wnt/β-catenin signaling pathway contributes to the development of low magnesium-induced OA. The current study was designed to investigate the effect of low dietary magnesium intake on OA development in a destabilization of the medial meniscus (DMM) OA model and to determine whether autophagy and the Wnt/β-catenin signaling pathway were involved in magnesium deficiency induced anabolic and catabolic changes.

## Methods

### Mice and experimental design

This study was carried out in accordance with the welfare and ethical principles of laboratory animal. The experimental protocol was approved by the Laboratory Animal Welfare and Ethical Committee of Central South University.

In the present study, the C57BL/6J mice (8-week-old, forty, SJA Animal Laboratory) were kept in standard, specific pathogen-free facility at a controlled temperature (22–24 °C), under a 12 h dark/light cycle and fed with a basal control diet for four weeks before DMM surgery. The mice were then randomly divided into four groups (ten mice each group). One group received sham-operation, and the other three groups were subject to DMM surgery to induce OA. After surgery, the mice were kept under the same conditions and were fed diets with different content of magnesium for twelve weeks. The diets were a commercial pelleted diet purchased from an animal diet company (Trophic Animal Feed High-Tech Co., Ltd). The sham and one DMM group were fed a basal control diet with magnesium content of 500 mg/kg following the recommendations of the American Institute of Nutrition (AIN-93G) [31–33]. The other two DMM groups received low magnesium diet with magnesium content of 100 mg/kg [34] and 300 mg/kg [35] according to previous studies, respectively. The diet compositions were shown in Table 1.

**Table 1:** Experimental diet composition

Composition	Control diet	Low-magnesium diet
**Magnesium (mg/kg)**^a^	**500.00**^b^	**300.00 or 100.00**
**Other essential mineral elements (mg/kg)**		
Phosphorus	1561.00	
Potassium	3600.00	
Sulfur	300.00	
Sodium	1019.00	
Chloride	1571.00	
Calcium	5000.00	
Iron	35.00	
Zinc	30.00	
Manganese	10.00	
Copper	6.00	
Iodine	0.20	
Molybdenum	0.15	
Selenium	0.15	
**Potentially beneficial mineral elements (mg/kg)**^c^		
Silicon	5.00	
Chromium	1.00	
Fluoride	1.00	
Nickel	0.50	
Boron	0.50	
Lithium	0.10	
Vanadium	0.10	
**Common ingredients (g/kg)**		
Cornstarch	397.49	
Casein (≥ 85%protein)	200.00	
Dextrinized cornstarch	132.00	
Sucrose	100.00	
Soybean oil (no additives)	70.00	
Fiber	50.00	
Vitamin mix ^d^	10.00	
L-Cystine	3.00	
Choline bitartrate (41% choline)	2.50	
Tert-butylhydroquinone	0.014	

^a^A similar composition was used in all the experimental groups, except for the addition of MgO to provide (per kg) 500.00 mg of Mg in the control and 300.00 mg and 100.00 mg of Mg in low magnesium diets

^b^The control diet follows the recommendations of American Institute of Nutrition (AIN-93G)

^c^Although biochemical functions have not been described, and essentiality has not been firmly established for any of these elements, feeding diets with very low quantities of some of them may result in negative effects on growth, reproductive performance in a variety of animals

^d^Vitamin mixture expressed in per kg diet: Nicotinic acid, 30 mg; pantothenate, 15 mg; pyridoxine, 6 mg; thiamin, 5 mg; riboflavin, 6 mg; folic acid, 2 mg; vitamin K, 750 μg; biotin, 200 μg; vitamin B-12, 25 μg; vitamin A, 4000 IU; vitamin D3, 1000 IU; vitamin E, 75 IU

Mice were fed their respective diets and distilled water ad libitum and allowed to move freely within their cages after surgery. Fresh diets were changed twice a week. Body weight was measured weekly. After 12 weeks of surgery, the mice were sacrificed for histological evaluation.

### Experimental OA model

The osteoarthritis model was induced by DMM surgery as previously described [36]. Briefly, the knee joint capsule was opened with an incision just medial to the patellar tendon and the medial meniscotibial ligament was sectioned with microsurgical scissors. The sham group was performed the surgery of arthrotomy without the transaction of medial meniscotibial ligament. All the mice had the right knee performed the surgery.

### Histological assessment and immunohistochemistry

The knee joints were fixed in 4% paraformaldehyde at 4 °C for 24 h and decalcified in 15% ethylenediamine tetraacetic acid (EDTA) (G1105, Servicebio) for 2 weeks and then embedded in paraffin. The serial frontal section of the entire knee joint with a thickness of 5 μm were obtained by the micro-slicer (Leica), and then the slices were stained with Safranin O/Fast Green to evaluate the cartilage degeneration of tibia plateau and femoral condyle by Osteoarthritis Research Society International (OARSI) scoring system [37]. For each joint, all four tibiofemoral compartments, the medial tibial plateau (MTP), medial femoral condyle (MFC), lateral tibial plateau (LTP), and lateral femoral condyle (LFC), were scored. The maximum OARSI scores (the maximum score of four compartments) and summed scores (sum score of four compartments) were presented according to previous studies [38].

An immunohistochemistry (IHC) assay was measured with Histostain-Plus Kits (SP-9001, ZSGB-BIO). Briefly, knee joint sections were deparaffinized, peroxidase quenched, and performed antigen retrieval with pepsin. Then, the sections were blocked in normal goat serum and incubated with specific primary antibodies overnight at 4 °C (Beclin1, 1:200, ab217179, abcam; LC3-II, 1:200, ab43894, abcam; β-catenin, 1:200, ab227499, abcam; TRPM7, 1:500, ab245408, abcam). After incubating with secondary antibodies, sections were stained with DAB (ZLI-9018, ZSGB-BIO), and counterstained with hematoxylin.

### Culture of articular chondrocytes

Mouse articular chondrocytes were isolated from femoral head, femoral condyle, and tibial plateaus of C57BL/6J mice (5–6 days old, SJA Animal Laboratory) by the method of enzymatic digestion as previously described [39, 40]. The chondrocytes were cultured in Dulbecco’s modified Eagle’s medium/F12 (DMEM/F12) (C11330500BT, Gibco) containing 10% fetal bovine serum (FBS) (10099-141 Gibco) and 1% penicillin/streptomycin solution (15140122, Gibco) and then incubated at 37 °C with 5% CO_2_ in a plastic culture flask.

### Treatment of chondrocytes

In the present study, chondrocytes were plated in 6-well culture plates at a density of 1 × 10^6^/well and cultured in DMEM/F12 containing 10% FBS and 1% penicillin/streptomycin solution. For low magnesium treatment, 0.7 mM, concentration of magnesium in regular DMEM/F12 medium was deemed as normal magnesium, which is also within the clinical reference range for magnesium in normal human serum [41, 42]. Medium containing 0.1 mM and 0.4 mM magnesium were used as low magnesium conditions which mimic dietary magnesium deficiency in vitro according to previous studies [43, 44]; 5 ng/mL TNF-α (315-01A, PeproTech) was used to induce catabolic factors expression in chondrocytes. To determine whether low magnesium conditions have an impact on autophagy, cells were treated with 10 μM Rapamycin (9904S, Cell Signaling Technology) to induce autophagy activity. Next, blocking experiments were carried out to determine the possible involvement of Wnt/β-catenin signaling pathway in low magnesium-reduced autophagy. Chondrocytes were incubated in medium containing 10 μM Wnt pathway inhibitor XAV939 (S1180, Selleck) for 24 h prior to low magnesium and rapamycin treatment.

### RNA extraction and quantificational real-time polymerase chain reaction (qRT-PCR)

Total RNA was extracted from murine chondrocytes using TRIzol® reagent (15596026, Invitrogen). Briefly, the chondrocytes were lysed in TRIzol® reagent, phases were separated by chloroform (20%), and supernatant was removed. RNA was precipitated using 100% isopropanol (0.5%) and washed twice with 70% ethanol. Following air-drying, RNA was resuspended in RNAse free water. The concentration of each RNA sample was measured by spectrophotometer (BioTek). Complementary DNA (cDNA) synthesis was performed by 1 μg of total RNA using a cDNA synthesis kit (RR047B, TaKaRa) according to the manufacturer’s protocols. qRT-PCR assay was performed using an All-in-One™ qPCR Mix kit (QP001, genecopoeia), with the final volume of 10 μL including 5 μL 2x SYBR Green QPCR master mixes, 1 μL of each primer (10 mM), 0.2 μL diluted Reference Dye, and 1 μL cDNA with addition of 1.8 μL DNase/RNase-Free water. The thermal cycling conditions were pre-denaturation at 95 °C for 10 min, followed by 40 cycles of denaturation at 95 °C for 15 s, annealing at 60 °C for 30 s, and extension at 72 °C for 30 s. Results were obtained using ABI Prism 7700 sequence detection software and Comparative quantification was determined using the 2^−ΔΔCt^ method. According to previous study, two housekeeping genes *β-actin and Hprt*1 were used for studies with mouse chondrocytes [45]. The primers used in this study are listed in Table 2.

**Table 2:** Sequence of primers for qRT-PCR

Gene symbol	Forward (5′→3′)	Reverse (5′→3′)
*Col2a1*	AGCGACTGTCCCTCGGAAAAAC	CCAGGTAGGCGATGCTGTTCTTAC
*Acan*	CCTGCTACTTCATCGACCCC	AGATGCTGTTGACTCGAACCT
*Sox9*	TCAGATGCAGTGAGGAGCAC	CCAGCCACAGCAGTGAGTAA
*Mmp-13*	ACAGGCTCCGAGAAATGCAA	CCACATCAGGCACTCCACAT
*Wnt3a*	CCTCTCGGATACCTCTTAGTGC	AGCTGCTTCGGTACCAGG
*Wnt5a*	CTGTCTTTGGCAGGGTGATG	TAGTCGATGTTGTCTCCGCA
*Wnt5b*	TGTTCATCATTGGCGCTCAG	TACGTGAAGGCAGTCTCTCG
*Wnt16*	CCACTACCACTTCCACCCAG	CTGAACCACATGCCGTACTG
*Catenin*	TGATTCGAAACCTTGCCCTT	AGCAAGGATGTGGAGAGCTC
*β-actin*	GGCTGTATTCCCCTCCATCG	CCAGTTGGTAACAATGCCATGT
*Hprt1*	CTGGTGAAAAGGACCTCTCGAA	CTGAAGTACTCATTATAGTCAAGGGCAT

### Western blot

The chondrocytes were lysed in SDS reagent with protease inhibitor (1:100), and the protein concentration of the lysate was measured by BCA protein assay (23225, ThermoFisher Scientific). The protein was separated by 8–12% SDS-polyacrylamide gel electrophoresis (SDS-PAGE) (genescript) and transferred to polyvinylidene difluoride (PVDF) membranes. Following blocking in 5% (w/v) skimmed milk for 1 h, membranes were incubated overnight at 4 °C in primary antibodies against MMP-13 (1:1,000, ab39012, abcam), Beclin1 (1:1,000, 3738S, Cell Signaling Technology), LC3 (1:1,000, 3868S, Cell Signaling Technology), β-catenin (1:1,000, 8480S, Cell Signaling Technology), β-Tubulin (1:3,000, T0023, Affinity Biosciences), GAPDH (1:1,000, SC32233, Santa Cruz), and TATA-binding protein (TBP) (1:1,000, 8515S, Cell Signaling Technology) and then incubated with horseradish peroxidase (HRP)-conjugated anti-rabbit IgG (1:5,000, ab150077, abcam) for 1 h at room temperature. Immunoreactive proteins were visualized with enhanced chemiluminescence (ECL) (10300, NCM biotech). The signal intensity was qualified by Bio-Rad chemiDoc-XRS with image lab software (BioRad).

### Autophagy detection with DALGreen staining

The autolysosome was detected using the method of fluorescence assay described as previously published [46]. Briefly speaking, the total number of 150,000 cells were plated in 24-well plate and co-cultured at 37 °C with 250 μL of 1 μM DALGreen (D675, Dojindo molecular technologies) for 30 min. Then, the cells were washed twice with DMEM/F12 growth medium and cultured in the medium containing different concentrations of magnesium. The activation of autophagy was induced with 10 μM Rapamycin (9904S, Cell Signaling Technology). Subsequently, intracellular autolysosomes were observed by fluorescence microscope, and the number of autolysosomes per cell was blinded-counted by two experienced collaborators.

### Transmission electron microscopy (TEM)

Transmission electron microscopy (TEM) was performed to evaluate the autophagosomes of the chondrocytes cultured in different concentrations of magnesium. Briefly, chondrocytes were treated with rapamycin under different magnesium conditions, and then cells were harvested and fixed with 2.5% glutaraldehyde at 4 °C overnight, subsequently fixed at 1% osmium tetroxide for 2 h and dehydrated. Next, the samples were encapsulated and sliced into ultrathin slices. Finally, the ultrathin sections were imaged with TEM (HITACHI).

### Detection of intracellular Mg^2+^ in chondrocytes

Mag fura-2 AM was used as a fluorescent probe to measure intracellular Mg^2+^. Briefly, chondrocytes were cultured in the medium with different concentrations of magnesium and then co-cultured with 2 μM Mag-Fluo-2 (M1292, Thermo Scientific) and 1 μM F-127 (P2443, Sigma) for 30 min. Subsequently, chondrocytes were washed with Mg^2+^-free medium for three times and counterstained cell nucleus with 1 μM Hoechst 33342 (C1028, Beyotime Biotechnology). The chondrocytes were immediately transferred to detect fluorescence intensity by spectrophotometer (BioTek) and imaged by a fluorescence microscope (Leica).

### Statistical analysis

All statistical analyses were performed with the SAS software, version 9.4 (SAS Institute, Cary, North Carolina, USA). Data are expressed as the mean ± SD according to the suggestion in a previous study [47]. The statistical analysis of the differences between two experimental groups was performed by the unpaired Student’s *t*-test. Differences among groups were determined by one-way ANOVA test. A *P* value less than 0.05 was taken as statistically significant.

## Results

### Dietary magnesium deficiency aggravated DMM-induced articular cartilage damage

**Fig. 1:**
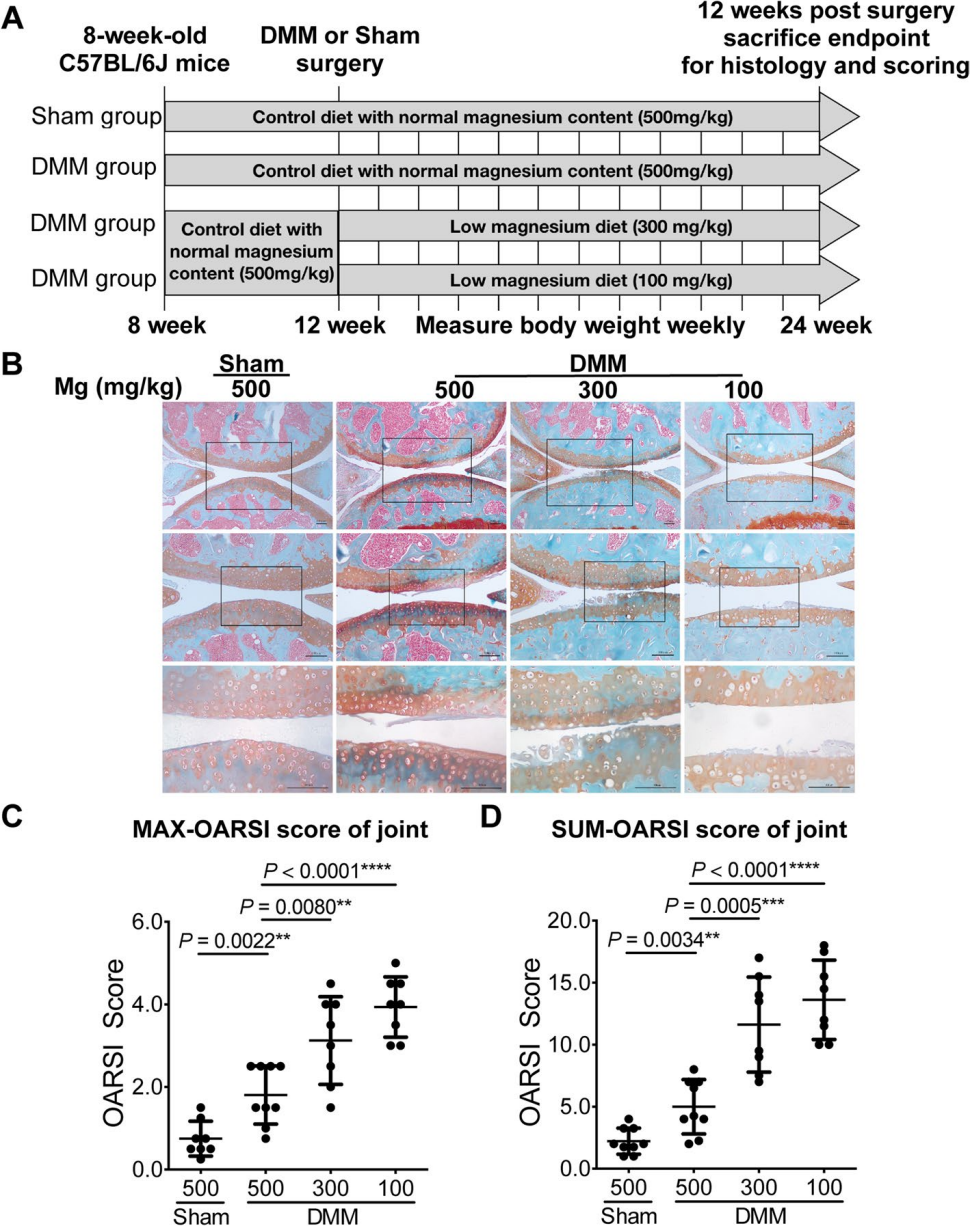
Dietary magnesium deficiency aggravated DMM - induced articular cartilage damage. **A** Schematic representation of in vivo experimental protocols. **B** Representative images of Safranin O/Fast Green–stained sections of knee joints from sham and DMM-induced OA mice fed with diets containing different amounts of magnesium (500 mg/kg, 300 mg/kg, 100 mg/kg). The low magnesium groups presented with severe cartilage damage and less Safranin O staining. Magnified images of regions marked by black boxes. Scale bar: 100 μm. **C**, **D** The severity of articular cartilage damage of mice groups above was quantified with OARSI scoring. The maximum OARSI scores (the maximum score of four compartments) and summed scores (sum score of four compartments) were presented (*n* = 9). Data were expressed as the mean ± SD and analyzed by one-way ANOVA test. (*P< 0.05, **P< 0.01, ***P< 0.001, ****P< 0.0001)

To examine the effect of dietary magnesium deficiency in DMM-induced OA mouse model, mice were subjected to DMM-surgery and fed low magnesium diet (300 mg/kg and 100 mg/kg) or basal control diet (magnesium concentration is 500 mg/kg). Mice subjected to sham-surgery were fed a basal control diet (Fig. 1A). After treatment for 12 weeks, there was no change in the body weight of any group (Supplementary Fig. 1). Histopathological analyses showed severe cartilage damage and loss of proteoglycans in low magnesium diet groups, compared with basal control diet group (Fig. 1B). The OARSI score among the two low magnesium diet groups were both significantly higher than control groups in DMM mouse model with a dose-dependent manner (Fig. 1C, D): MAX-OARSI score: 1.81 ± 0.70 (500 mg/kg) vs 3.13 ± 1.06 (300 mg/kg) vs 3.94 ± 0.73 (100 mg/kg); SUM-OARSI score: 5.00 ± 2.19 (500 mg/kg) vs 11.63 ± 3.83 (300 mg/kg) vs 13.63 ± 3.20 (100 mg/kg), indicating that dietary magnesium deficiency aggravated OA progression of mouse model.

### Reduced autophagy contributed to pro-catabolic and anti-anabolic effects in chondrocytes after the exposure to magnesium deficient conditions

**Fig. 2:**
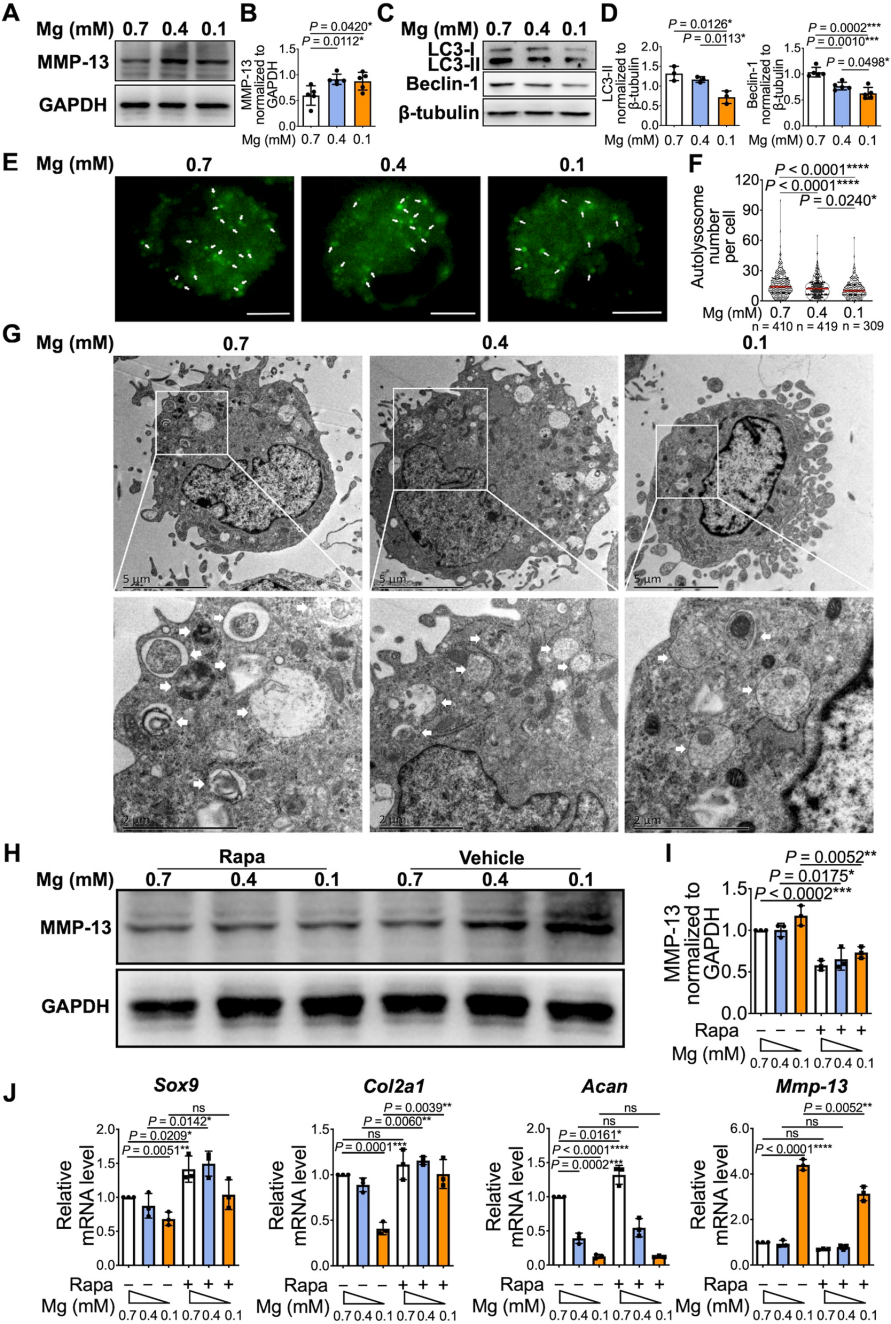
Reduced autophagy contributed to pro-catabolic and anti-anabolic effects after the exposure to magnesium deficient conditions. **A** Mouse chondrocytes were cultured with TNF-α under different magnesium conditions (0.7 mM, 0.4 mM, and 0.1 mM). Protein levels of MMP13 was detected by western blot. **B** The quantification of protein expression of MMP13 was done by densitometry analysis of the protein bands. Values were normalized to GAPDH (*n* = 5). **C** Protein level of LC3-II and Beclin-1 under different magnesium conditions were analyzed by western blot. **D** The quantification of protein expression of LC3-II and Beclin-1 was done by densitometry analysis of the protein bands. Values were normalized to β-tubulin (*n* = 5). **E** Chondrocyte imaging with DALGreen staining under different magnesium conditions. Autolysosomes were marked with white arrows. Scale bar: 5 μm. **F** Number of autolysosomes per cell was quantitated. **G** TEM analysis of autophagosomes in chondrocytes under different magnesium conditions. Autophagosomes were marked with white arrows. Scale bar: 5 μm. **H** Mouse chondrocytes were cultured with TNF-α under different magnesium conditions with or without rapamycin (Rapa) pretreatment. Protein levels of MMP13 was detected by western blot. **I** The quantification of protein expression of MMP13 was done by densitometry analysis of the protein bands. Values were normalized to GAPDH (*n* = 3). (J) *Sox9*, *Col2a1*, *Acan*, and *Mmp-13* genes expression were assessed by qRT-PCR (*n* = 3). *β-actin* was used as housekeeping gene. Data were expressed as the mean ± SD and analyzed by one-way ANOVA test. (*P< 0.05, **P< 0.01, ***P< 0.001, ****P< 0.0001)

To gain insights into the underlying mechanism, we further investigated whether autophagy is involved in magnesium deficiency-induced OA progression. We first verified changes of anabolic and catabolic factors in response to low magnesium conditions in mouse chondrocytes. The protein level of matrix metalloproteinase 13 (MMP-13) increased in response to low magnesium conditions at 0.1 mM and 0.4 mM (Fig. 2A, B), while low magnesium at 0.1 mM decreased the mRNA level of anabolic genes *Sox9*, *Col2a1*, and *Acan*, compared with the normal magnesium group (0.7 mM magnesium concentration in regular DMEM/F12 medium) (Fig. 2J and Supplementary Fig. 2). Meanwhile, low magnesium suppressed the expression of autophagy markers LC3-II and Beclin-1 (Fig. 2C, D), which is indicative of defective autophagy. Consistent with the in vitro study, the expression of LC3-II and Beclin-1 in cartilage was also reduced in mice fed with the magnesium deficient diet as determined by IHC staining (LC3-II positive cells (%): 37.04 ± 2.15 (500 mg/kg) vs 28.04 ± 3.29 (300 mg/kg) vs 23.03 ± 4.38 (100 mg/kg); Beclin-1 positive cells (%): 33.97 ± 4.81 (500 mg/kg) vs 18.34 ± 3.71 (300 mg/kg) vs 16.81 ± 3.83 (100 mg/kg)) (Supplementary Fig. 3 and 4).

To further examine the autophagy activity, chondrocytes were incubated with DALGreen, a small green hydrophobic molecule used for autolysosome imaging. The autolysosomes were significantly decreased in low concentrations of magnesium at 0.1 mM and 0.4 mM, compared to normal magnesium level: 11.50 ± 8.31 (0.1 mM) vs 12.95 ± 9.14 (0.4 mM) vs 16.07 ± 11.05 (0.7 mM) (Fig. 2E, F). Less autophagosomes under low magnesium conditions were also observed by transmission electron microscopy (Fig. 2G).

Then, we used rapamycin, an activator of autophagy, to determine the effects of autophagy activation on magnesium deficiency induced changes in anabolic and catabolic factors. Rapamycin significantly attenuated low magnesium stimulated *Mmp-13* mRNA and protein expression (Fig. 2H, I, J and Supplementary Fig. 2) and restored the inhibitory effect of low magnesium on *Sox9*, *Col2a1*, and *Acan* gene expression (Fig. 2J and Supplementary Fig. 2), suggesting that the anabolic inhibitory and catabolic stimulatory effects due to magnesium deficiency were mediated by reduced autophagy.

### Magnesium deficiency activated Wnt/β-catenin signaling in chondrocytes

**Fig. 3:**
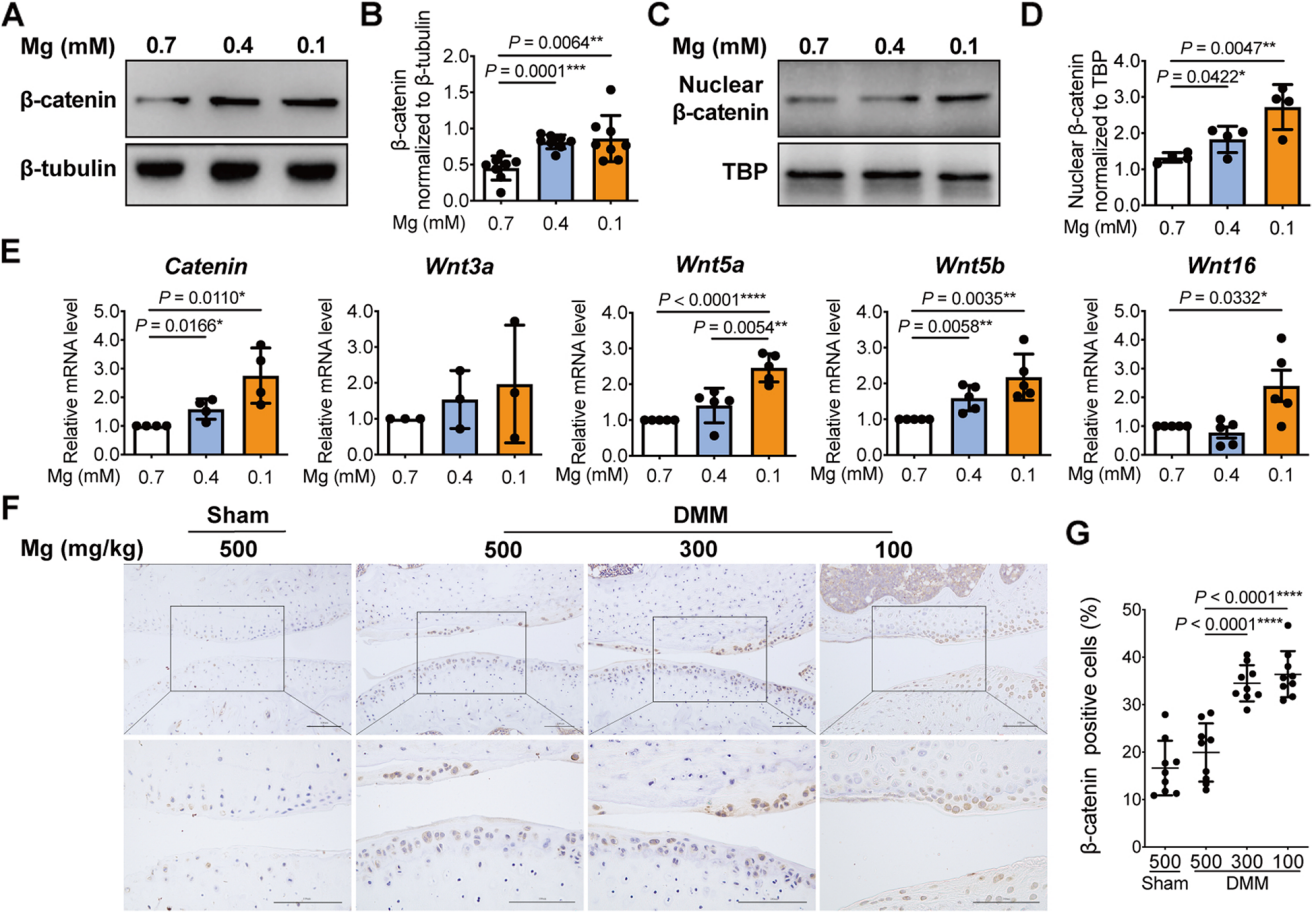
Magnesium deficiency activated Wnt/β-catenin signaling in chondrocytes. Mouse chondrocytes were cultured under different magnesium conditions (0.7 mM, 0.4 mM and 0.1 mM). Protein levels of β-catenin in total cell lysates (**A**, **B**) and nuclear fractions (**C**, **D**) were detected by western blot. The quantitation of protein expression of β-catenin was done by densitometry analysis of the protein bands. Values were normalized to β-tubulin and TATA-binding protein (TBP), respectively (*n* = 8 for β-catenin in total cell lysates; *n* = 4 for nuclear β-catenin). **E** mRNA levels of *Catenin*, *Wnt3a*, *Wnt5a*, *Wnt5b*, and *Wnt16* were investigated by qRT-PCR (*n* = 3, 4 or 5). *β-actin* was used as housekeeping gene. **F**, **G** Immunostaining and quantitative analysis of cells positive for β-catenin in sham and DMM-induced OA mice fed with diets containing different amounts of magnesium (500 mg/kg, 300 mg/kg, 100 mg/kg) (*n* = 9). Scale bar: 100 μm. Data were expressed as the mean ± SD and analyzed by one-way ANOVA test. (*P< 0.05, **P< 0.01, ***P< 0.001, ****P< 0.0001)

To identify the magnesium deficiency-activated signaling pathway that is responsible for reduced autophagy, we examined the Wnt/β-catenin signaling pathway in magnesium deficient chondrocytes. We found that the expression of the core factor of the Wnt/β-catenin signaling pathway, β-catenin, was upregulated under low magnesium conditions at both the mRNA and protein levels (Fig. 3A, B, E). Because β-catenin translocates to the nucleus and regulates its target gene expression when the Wnt pathway is activated, we further separated the nuclear and cytosolic fractions of chondrocytes cultured in medium containing different concentrations of magnesium. Immunoblot results showed magnesium concentrations at both 0.1 mM and 0.4 mM increased β-catenin levels in the nucleus (Fig. 3C, D). Then, mRNA levels of representative Wnt ligands were examined. The expression of *Wnt5a*, *Wnt5b*, and *Wnt16* were significantly increased in response to low magnesium conditions, while *Wnt3a* showed a similar trend with no statistical significance when using *β- actin* as housekeeping gene, and its expression level was very low in primary mouse chondrocytes, indicated by high ΔCt values, which was consistent with a previous study [48] (Fig. 3E, Supplementary Fig. 5 and Supplementary Table 1). Increased cartilage expression of β-catenin induced by low dietary magnesium intake was also verified in mice by IHC (β-catenin positive cells (%): 19.92 ± 6.13 (500 mg/kg) vs 34.51 ± 3.81 (300 mg/kg) vs 36.43 ± 4.86 (100 mg/kg)) (Fig. 3F, G). These findings demonstrate an activation of the Wnt/β-catenin signaling pathway induced by magnesium deficiency.

### Activation of Wnt/β-catenin signaling contributed to reduction in autophagy

**Fig. 4:**
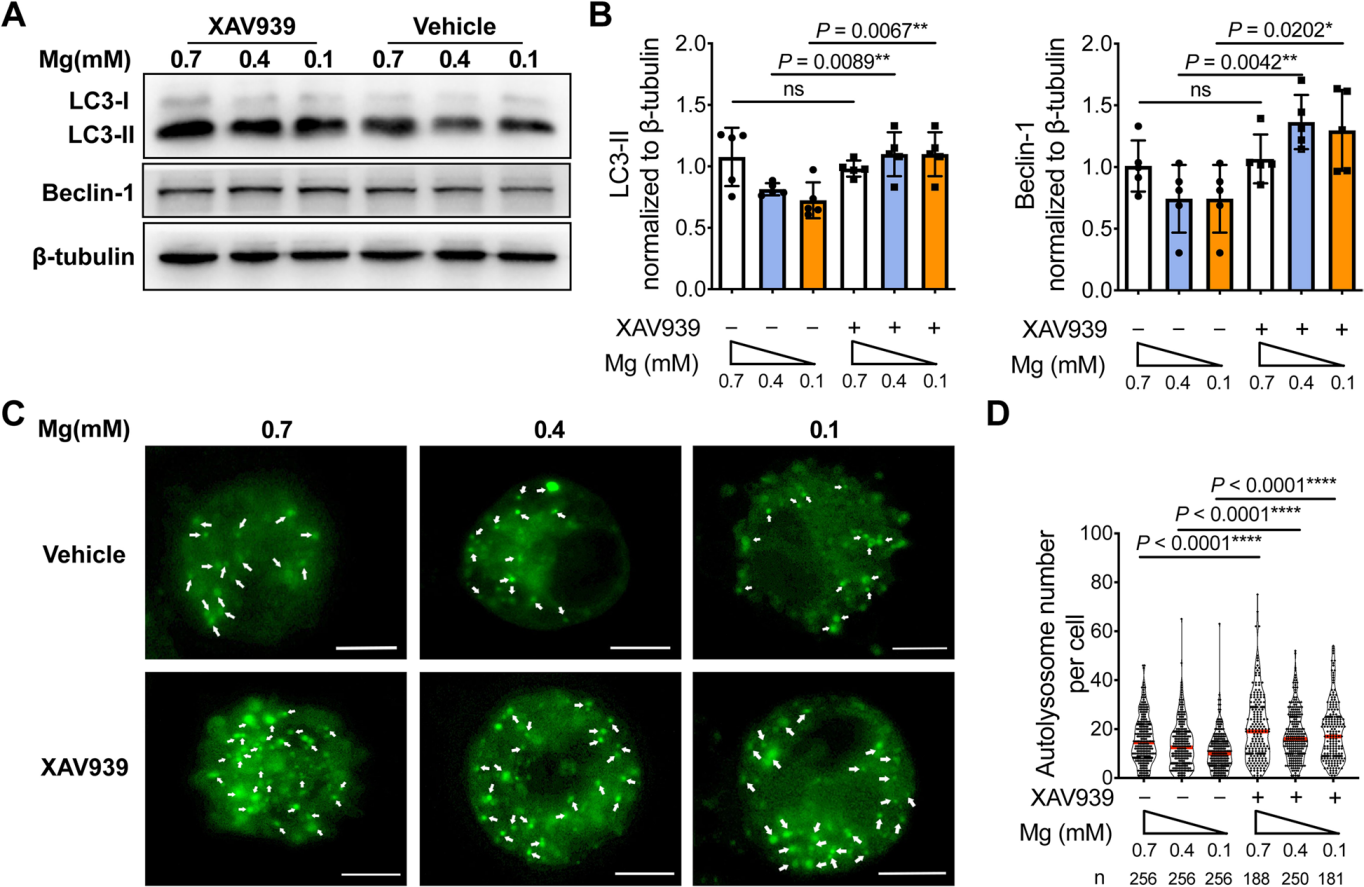
Activation of Wnt/β-catenin signaling contributed to reduction in autophagy. **A** Mouse chondrocytes were cultured under different magnesium conditions with or without XAV939 pretreatment. Protein levels of LC3-II and Beclin-1 under different magnesium conditions were analyzed by western blot. **B** The quantification of protein expression of LC3-II and Beclin-1 was done by densitometry analysis of the protein bands. Values were normalized to β-tubulin (*n* = 5). **C** Chondrocyte imaging with DALGreen staining. Autolysosomes were marked with white arrows. Scale bar: 5 μm. **D** Number of autolysosomes per cell was quantified. Data were expressed as the mean ± SD and analyzed by one-way ANOVA test. (*P< 0.05, **P< 0.01, ***P< 0.001, ****P< 0.0001)

To investigate whether the Wnt/β-catenin signaling pathway is involved in the autophagy inhibition due to a magnesium deficiency, XAV939, a Wnt/β-catenin signaling pathway inhibitor, which facilitates β-catenin degradation, was used. When XAV939 was added, significantly higher expression levels of LC3-II and Beclin-1 were found, compared to the control group under the same concentration of magnesium (Fig. 4A, B). Likewise, autolysosomes labeled with DALGreen in chondrocytes under low magnesium conditions at 0.1 mM and 0.4 mM treated by the XAV939 also increased compared to those with vehicle treatment (0.1 mM: 18.99 ± 12.11 (XAV939) vs 10.79 ± 7.79 (vehicle); 0.4 mM: 17.34 ± 10.31 (XAV939) vs 13.49 ± 9.77 (vehicle)) (Fig. 4C, D). Hence, reduced autophagy caused by a magnesium deficiency can be restored to normal levels by inhibition of Wnt/β-catenin signaling pathway.

### Impaired magnesium homeostasis altered TRPM7 expression

**Fig. 5:**
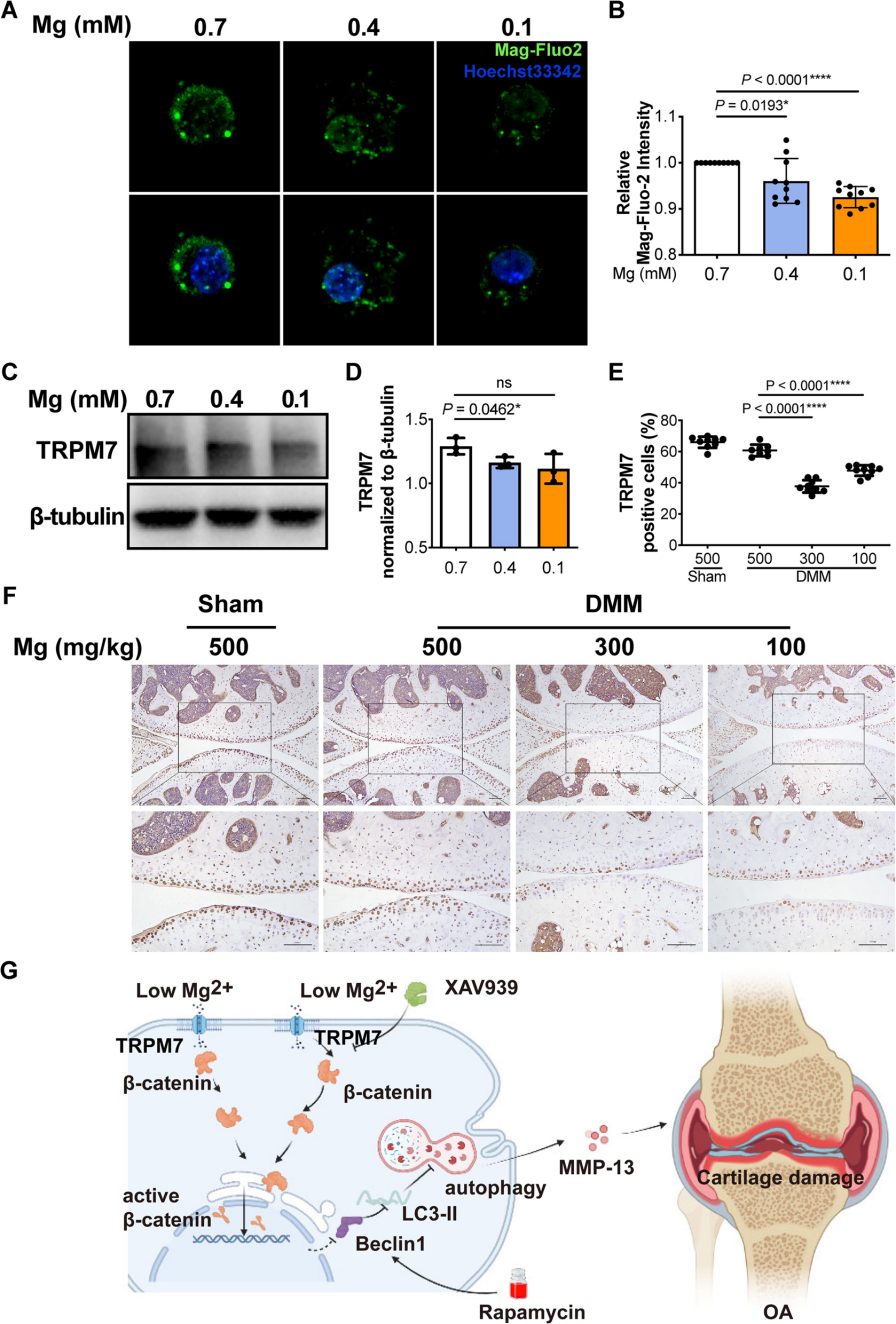
Impaired magnesium homeostasis induced by magnesium deficiency was mediated by TRPM7. **A** Representative fluorescence images showing the concentration of intracellular Mg^2+^ of chondrocytes cultured under different magnesium conditions (0.7 mM, 0.4 mM, and 0.1 mM) for 48 h. **B** Intracellular Mg^2+^ concentration quantified by measuring the intensity of fluorescence (*n* = 10). **C** Protein level of TRPM7 under different magnesium conditions for 48 h were analyzed by western blot. **D** The quantification of protein expression of TRPM7 was done by densitometry analysis of the protein bands. Values were normalized to β-tubulin (*n* = 3). **E**, **F** Immunostaining and quantitative analysis of cells positive for TRPM7 in sham and DMM-induced OA mice fed with diets containing different amounts of magnesium (500 mg/kg, 300 mg/kg, 100 mg/kg) (*n* = 9). Scale bar: 100 μm. Data were expressed as the mean ± SD and analyzed by one-way ANOVA test (*P< 0.05, **P< 0.01, ***P< 0.001, ****P< 0.0001). **G** Summary of the present study: Extracellular magnesium deficiency downregulates the expression of TRPM7, resulting in reduced intracellular magnesium. Subsequently, intracellular magnesium deficiency inhibits autophagy of chondrocytes by the activation of the Wnt/β-catenin signaling pathway and thus aggravates cartilage damage

To explore how extracellular magnesium deficiency affected magnesium homeostasis, we studied whether low magnesium concentrations changed the influx of Mg^2+^ in chondrocytes. Using Mag-Fluo-2, an Mg^2+^ specific dye, we observed a decrease of intracellular Mg^2+^ levels under extracellular low magnesium conditions for 48 h (Fig. 5A, B). Expression of TRPM7, an essential channel regulating the cellular influx of Mg^2+^, significantly decreased under extracellular low magnesium conditions (Fig. 5C, D). In line with these observations, decreased cartilage expression of TRPM7 in dietary magnesium deficient mice was also observed by IHC (TRPM7 positive cells (%): 60.81 ± 3.71 (500 mg/kg) vs 37.76 ± 3.97 (300 mg/kg) vs 47.82 ± 3.41 (100 mg/kg)) (Fig. 5E, F). These results indicated that magnesium deficiency decreased intracellular Mg^2+^ level by suppressing the expression of TRPM7, resulting in disrupted magnesium homeostasis, thus further activating signaling pathway that is associated with magnesium.

## Discussion

The present study established the role that dietary magnesium deficiency plays in the progression of experimental OA in mice. It also proposed a mechanism in which low magnesium conditions inhibited the autophagy of chondrocytes by activating the Wnt/β-catenin signaling pathway and consequently decreasing the expression of anabolic factors and increasing the expression of catabolic factors in chondrocytes. Furthermore, this pathological effect could be alleviated by rapamycin, a potent inducer of autophagy (Fig. 5G).

A growing body of evidence has demonstrated that dietary and blood magnesium deficiency is highly relevant to the development of radiographic OA [7, 9–12]. However, there was also another study reporting no significant association between dietary magnesium intake and clinical diagnosed OA [49]. Therefore, a causal relationship between magnesium deficiency and OA is worthy of further study. In addition to human observational studies, several animal studies examined the therapeutic effect of intra-articular administration of magnesium against osteoarthritis recently [50–52]. Yao et al. found intra-articular injection of magnesium chloride could attenuate the progression of experimental OA [51]. The protective effect was mainly mediated by promoting articular cartilage matrix synthesis and inhibiting synovial inflammation, and this effect can be enhanced by addition of vitamin C [50]. Our study focused on dietary magnesium deficient and showed that low dietary magnesium intake aggravated articular cartilage damage in mouse OA model. In the meantime, increase catabolic activity and decrease matrix synthesis were also observed in low magnesium treated chondrocytes. These findings suggested the essential role of adequate magnesium in daily diet for OA patient and those at high risk of OA.

Autophagy, which is an important cellular self-protection process, has been reported to be dependent on magnesium. However, the results regarding the effect of magnesium on autophagy are sometimes contradictory [19, 21, 53, 54]. Sun et al. showed that increased external Mg^2+^ concentration promoted expression of autophagy marker LC3 in dopaminergic neurons [54]. Castiglioni et al. also found that magnesium triggered autophagy via Akt-mTOR signaling in bladder cancer cells [53]. In contrast, there were other two studies showing an autophagy activation via low extracellular magnesium treatment and magnesium channel silence in bone marrow mesenchymal stem cells [19, 21]. Accordingly, these previous results indicated that whether autophagy is promoted or inhibited by magnesium was cell type dependent. In this study, we found that the expression of Beclin-1 and LC3-II was decreased both in low magnesium treated chondrocytes and low magnesium diet fed mice. Fewer autolysosomes and autophagosomes were also observed under low magnesium conditions by DALGreen staining and TEM, indicating magnesium deficiency inhibited autophagy of chondrocytes. In addition, recent studies have shown that the autophagy activity in OA decreased with aging [55] and administration of the autophagy activator, rapamycin, exerted a protective effect on OA [17, 18], while autophagy inhibitor aggravated the severity of experimental OA [56]. Based on the above evidence, we demonstrated that OA phenotypes induced by low magnesium conditions could be restored by rapamycin in chondrocytes, suggesting that magnesium deficiency promoted OA development by inhibiting autophagy.

Furthermore, we found that magnesium deficiency could activate the Wnt/β-catenin signaling pathway in chondrocytes via both in vivo and in vitro experiments. Wnt/β-catenin signaling cascades play an essential role in the development and metabolism of articular cartilage. Abnormal activation of Wnt/β-catenin signaling in chondrocytes induced articular cartilage calcification and osteophyte formation, inhibited anabolism, and promoted catabolism, finally resulting in OA [24, 27, 57, 58]. Meanwhile, Wnt/β-catenin signaling pathway activation could also inhibit autophagy activity while blocking or suppressing Wnt/β-catenin pathway may do the opposite [59, 60]. Thus, magnesium deficiency may activate the Wnt/β-catenin signaling pathway and subsequently inhibit autophagy. In the present study, we found that Wnt/β-catenin signaling pathway inhibitor, XAV939, negated the decrease of autophagy markers and autolysosomes induced by low magnesium conditions, demonstrating an important role of the Wnt/β-catenin signaling pathway in magnesium deficiency induced inhibition of autophagy in chondrocytes.

TRPM7 is an essential ion channel that is central in modulating Mg^2+^ homeostasis. It plays an important role in various cellular physiological process, like proliferation, and apoptosis [54, 61]. Several studies have reported that TRPM7 could mediate activation or inhibition of intracellular signaling pathways in response to a change of extracellular Mg^2+^ concentration [54, 61–65]. A recent study observed that an increase in extracellular Mg^2+^ contributed to activation of the NF-κB signaling pathway via upregulation of TRPM7 expression [65]. Similarly, Sun et al. also showed that an extracellular magnesium supplement increased TRPM7 expression and intracellular Mg^2+^, thus attenuating neurotoxin-induced apoptosis in dopaminergic neurons [54]. In addition, another study found that increasing of Mg^2+^ influx via TRPM7 prevented phosphate-induced calcification in vascular smooth muscle cells, which was mediated by the inhibition of the Wnt/β-catenin signaling pathway [63]. Therefore, consistent with the previous results above, in the present study, a decrease in the intracellular Mg^2+^ concentration was observed under low external Mg^2+^ conditions in chondrocytes. However, the previous studies above only reported the effect of extracellular Mg^2+^ on TRPM7 expression in in vitro experiments. Our study provided new evidence that the expression of TRPM7 is also greatly reduced in low magnesium diet fed mice, indicating a decreased level of magnesium in dietary deficient mice cartilage. These findings suggested that a decrease in TRPM7 level upon magnesium deficiency would contribute to altered Mg^2+^ homeostasis thereby leading to changes in signaling pathways.

## Conclusions

This study identified an essential role of dietary magnesium in the development of OA. Magnesium deficiency aggravated cartilage damage in a DMM-induced OA model, which was possibly mediated by reduced autophagy through the activation of the Wnt/β-catenin signaling pathway. These findings indicated potential benefits of adequate magnesium intake in the daily diet for OA patients or those individuals at high risk of OA. This also placed the Wnt/β-catenin signaling pathway and autophagy as potential therapeutic targets for OA treatment.

## Supplementary Information

**Additional file 1: Supplemental Figure 1**. Body weight of mice over time. Body weight of sham and DMM-induced OA mice fed with diets containing different content of magnesium (500 mg/kg, 300 mg/kg, 100 mg/kg) measured weekly. No statistical significance was observed between different groups at the same age.

**Additional file 2: Supplemental Figure 2**. *Sox9*, *Col2a1*, *Acan* and *Mmp-13* genes expression. Mouse chondrocytes were cultured under different magnesium conditions (0.7mM, 0,4mM and 0.1mM) with or without treatment of Rapa. *Sox9*, *Col2a1*, *Acan* and *Mmp-13* genes expression were assessed by qRT-PCR (n = 5). *Hprt1* was used as housekeeping gene. Data were expressed as the mean ± SD and analyzed by one-way ANOVA test. (^*^P<0.05, ^**^P<0.01, ^***^P<0.001, ^****^P<0.0001)

**Additional file 3: Supplemental Figure 3**. Immunohistochemistry analysis of LC3-II. (A, B) Immunostaining and quantitative analysis of cells positive for LC3-II in sham and DMM-induced OA mice fed with diets containing different content of magnesium (500 mg/kg, 300 mg/kg, 100 mg/kg) (n = 9). Scale bar: 100 μm. Data were expressed as the mean ± SEM and analyzed by one-way ANOVA test (*P<0.05, **P<0.01, ***P<0.001, ****P<0.0001).

**Additional file 4: Supplemental Figure 4**. Immunohistochemistry analysis of Beclin-1. (A, B) Immunostaining and quantitative analysis of cells positive for Beclin-1 in sham and DMM-induced OA mice fed with diets containing different content of magnesium (500 mg/kg, 300 mg/kg, 100 mg/kg) (n = 9). Scale bar: 100 μm. Data were expressed as the mean ± SEM and analyzed by one-way ANOVA test (*P<0.05, **P<0.01, ***P<0.001, ****P<0.0001).

**Additional file 5: Supplemental Figure 5**. *Catenin*, *Wnt3a*, *Wnt5a*, *Wnt5b* and *Wnt16* genes expression. Mouse chondrocytes were cultured under different magnesium conditions (0.7mM, 0,4mM and 0.1mM). mRNA levels of *Catenin*, *Wnt3a*, *Wnt5a*, *Wnt5b* and *Wnt16* were investigated by qRT-PCR (n = 5). *Hprt1* was used as housekeeping gene. Data were expressed as the mean ± SD and analyzed by one-way ANOVA test. (^*^P<0.05, ^**^P<0.01, ^***^P<0.001, ^****^P<0.0001).

**Additional file 6: Supplemental Table 1**. Ct values of Wnt ligands normalized to *β-actin* in qRT-PCR.

## Data Availability

The datasets analyzed during the current study are available from the corresponding author on reasonable request.

## Abbreviations

OA: Osteoarthritis
Mg: Magnesium
DMM: Destabilization of the medial meniscus
OARSI: Osteoarthritis Research Society International
TEM: Transmission electron microscopy
LC3: Microtubule associated protein 1 light chain 3
AIN: American Institute of Nutrition
TRPM7: Transient receptor potential cation channel subfamily M member 7
EDTA: Ethylenediamine tetraacetic acid
MTP: Medial tibial plateau
MFC: Medial femoral condyle
LTP: Lateral tibial plateau
LFC: Lateral femoral condyle
IHC: Immunohistochemistry
TNF-α: Tumor necrosis factor
qRT-PCR: Quantitative real-time polymerase chain reaction
DAB: Diaminobenzidine
DMEM/F12: Dulbecco’s modified Eagle’s medium/F12
FBS: Fetal bovine serum
cDNA: Complementary DNA
SDS: Sodium dodecyl sulfate
SDS-PAGE: SDS-polyacrylamide gel electrophoresis
PVDF: Polyvinylidenedifluoride
HRP: Horseradish peroxidase
MMP-13: Matrix metalloproteinase 13
*Col2a1*: Type II collagen alpha 1 chain
*Sox9*: SRY-box transcription factor 9
*Acan*: Aggrecan
Wnt: Wingless/integrated
Akt-mTOR: Akt-mechanistic target of rapamycin kinase
SD: Standard deviation
NF-κB: Nuclear factor kappa B
GADPH: Glyceraldehyde 3-phosphate dehydrogenase
TBP: TATA-box binding protein
ECL: Enhanced chemiluminescence
SD: Standard deviation
ANOVA: One-way analysis of variation
Ct: Comparative threshold cycle
Rapa: Rapamycin
