# Supplementary figures and images for: Increased Wnt/β-catenin signaling contributes to autophagy inhibition resulting from a dietary magnesium deficiency in injury-induced osteoarthritis
Source: Arthritis Res Ther. 2022 Jul 8;24:165. doi: 10.1186/s13075-022-02848-0 (PMC9264717; doi:10.1186/s13075-022-02848-0)

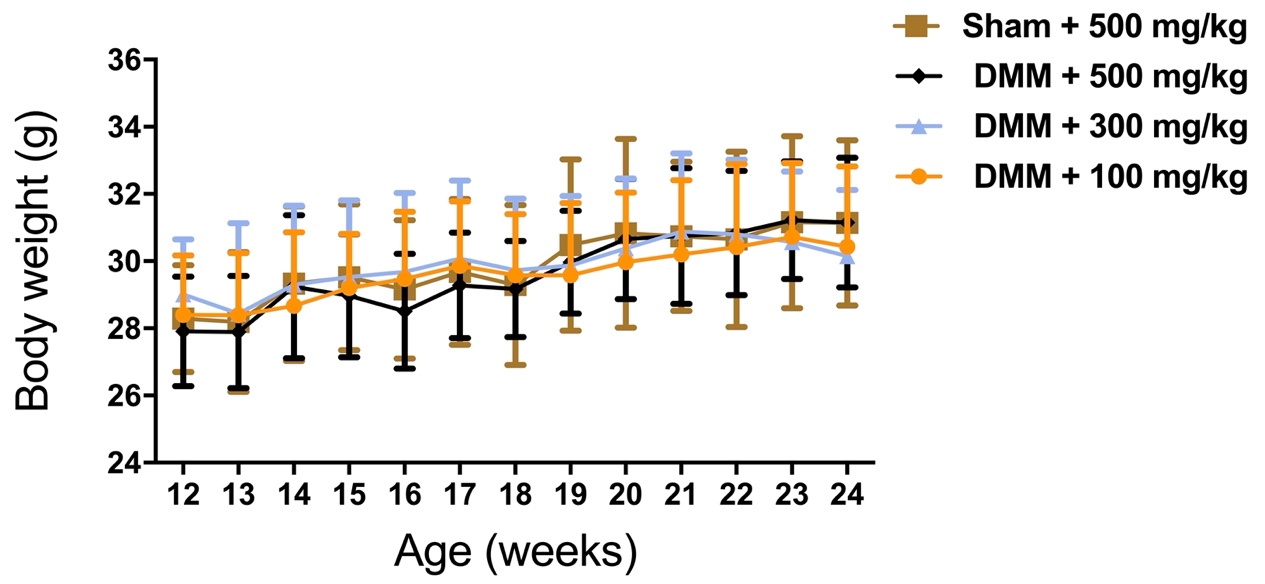

Supplement: Supplementary file 1 — Additional file 1: Supplemental Figure 1. Body weight of mice over time. Body weight of sham and DMM-induced OA mice fed with diets containing different content of magnesium (500 mg/kg, 300 mg/kg, 100 mg/kg) measured weekly. No statistical significance was observed between different groups at the same age. [file 13075_2022_2848_MOESM1_ESM.docx]

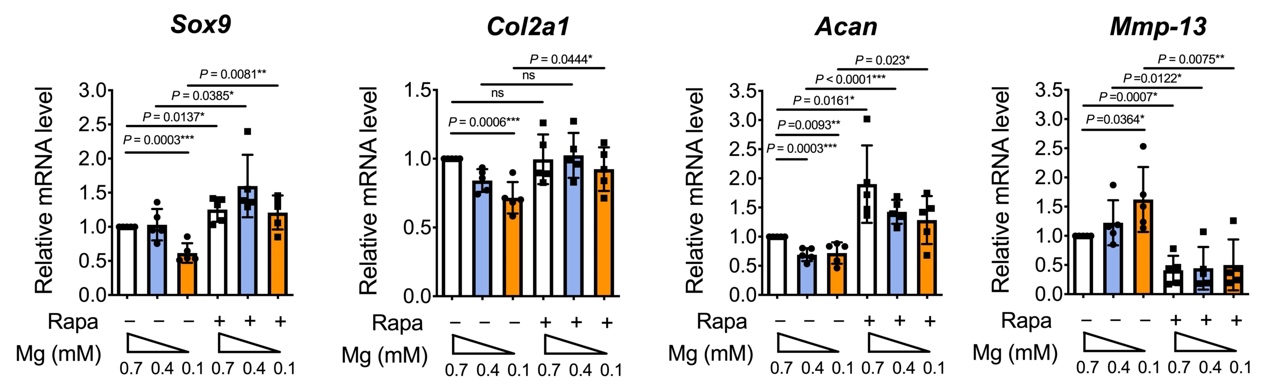

Supplement: Supplementary file 2 — Additional file 2: Supplemental Figure 2. Sox9, Col2a1, Acan and Mmp-13 genes expression. Mouse chondrocytes were cultured under different magnesium conditions (0.7mM, 0,4mM and 0.1mM) with or without treatment of Rapa. Sox9, Col2a1, Acan and Mmp-13 genes expression were assessed by qRT-PCR (n = 5). Hprt1 was used as housekeeping gene. Data were expressed as the mean ± SD and analyzed by one-way ANOVA test. (*P<0.05, **P<0.01, ***P<0.001, ****P<0.0001) [file 13075_2022_2848_MOESM2_ESM.docx]

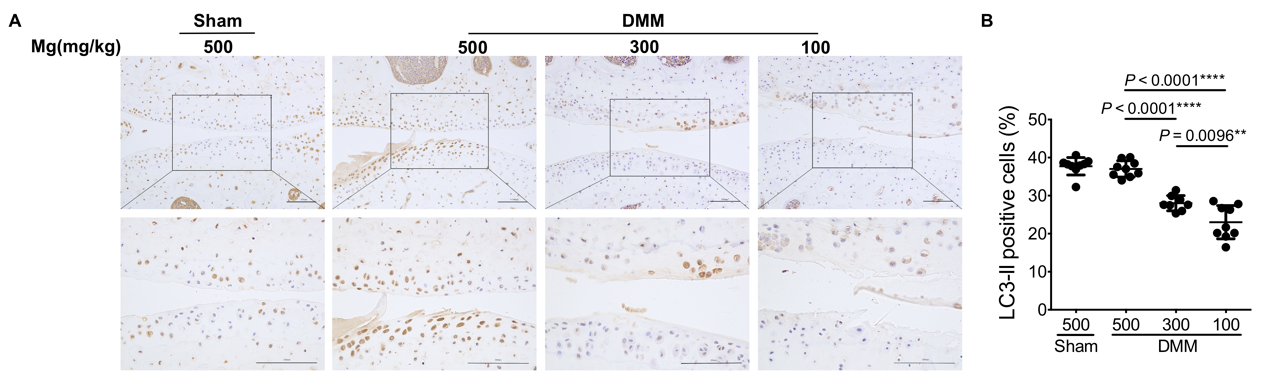

Supplement: Supplementary file 3 — Additional file 3: Supplemental Figure 3. Immunohistochemistry analysis of LC3-II. (A, B) Immunostaining and quantitative analysis of cells positive for LC3-II in sham and DMM-induced OA mice fed with diets containing different content of magnesium (500 mg/kg, 300 mg/kg, 100 mg/kg) (n = 9). Scale bar: 100 μm. Data were expressed as the mean ± SEM and analyzed by one-way ANOVA test (*P<0.05, **P<0.01, ***P<0.001, ****P<0.0001). [file 13075_2022_2848_MOESM3_ESM.docx]

**
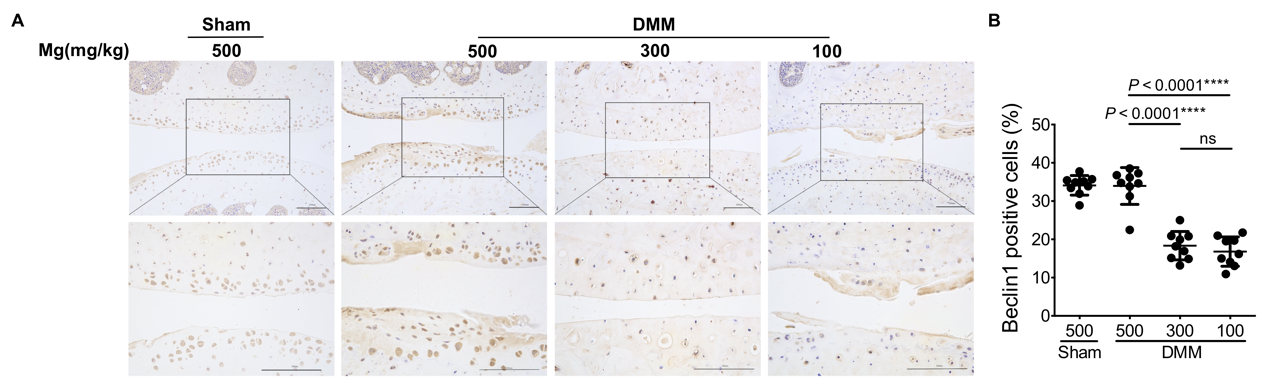
**

Supplement: Supplementary file 4 — Additional file 4: Supplemental Figure 4. Immunohistochemistry analysis of Beclin-1. (A, B) Immunostaining and quantitative analysis of cells positive for Beclin-1 in sham and DMM-induced OA mice fed with diets containing different content of magnesium (500 mg/kg, 300 mg/kg, 100 mg/kg) (n = 9). Scale bar: 100 μm. Data were expressed as the mean ± SEM and analyzed by one-way ANOVA test (*P<0.05, **P<0.01, ***P<0.001, ****P<0.0001). [file 13075_2022_2848_MOESM4_ESM.docx]

**
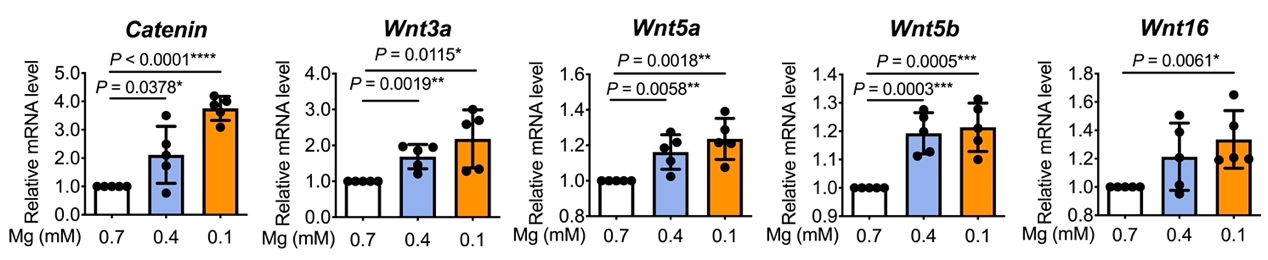
**

Supplement: Supplementary file 5 — Additional file 5: Supplemental Figure 5. Catenin, Wnt3a, Wnt5a, Wnt5b and Wnt16 genes expression. Mouse chondrocytes were cultured under different magnesium conditions (0.7mM, 0,4mM and 0.1mM). mRNA levels of Catenin, Wnt3a, Wnt5a, Wnt5b and Wnt16 were investigated by qRT-PCR (n = 5). Hprt1 was used as housekeeping gene. Data were expressed as the mean ± SD and analyzed by one-way ANOVA test. (*P<0.05, **P<0.01, ***P<0.001, ****P<0.0001). [file 13075_2022_2848_MOESM5_ESM.docx]
