# Supplementary material for: Increased Wnt/β-catenin signaling contributes to autophagy inhibition resulting from a dietary magnesium deficiency in injury-induced osteoarthritis
Source: Arthritis Res Ther. 2022 Jul 8;24:165. doi: 10.1186/s13075-022-02848-0 (PMC9264717; doi:10.1186/s13075-022-02848-0)
Supplement: Supplementary file 6 — Additional file 6: Supplemental Table 1. Ct values of Wnt ligands normalized to β-actin in qRT-PCR. [file 13075_2022_2848_MOESM6_ESM.docx]

| **Supplemental Table 1**  **Ct values of Wnt ligands normalized to *β-actin* in qRT-PCR** | | | |
| --- | --- | --- | --- |
| **Gene symbol** | **0.7 Mg(mmol/L)** | **0.4 Mg(mmol/L)** | **0.1 Mg(mmol/L)** |
| ***Wnt3a*** | 9.241 ± 2.6070 | 8.779 ± 1.8800 | 8.720 ± 1.4300 |
| ***Wnt5a*** | 8.084 ± 0.8937 | 7.698 ± 1.0130 | 6.801 ± 0.7796 |
| ***Wnt5b*** | 7.790 ± 1.2330 | 7.149 ± 0.9338 | 6.713 ± 0.9690 |
| ***Wnt16*** | 3.418 ± 1.1920 | 3.994 ± 0.7546 | 2.318 ± 1.7460 |
| Date shown: Mean ± SD | | | |
